# Biogeographic Structure and Mitonuclear Discordance Reveal Cryptic Diversity in Pacific Herring (*Clupea pallasii*)

**DOI:** 10.1002/ece3.72452

**Published:** 2025-11-21

**Authors:** Laura E. Timm, Sydney A. Almgren, J. Andrés López, Jessica R. Glass

**Affiliations:** ^1^ College of Fisheries and Ocean Sciences, University of Alaska Fairbanks Fairbanks Alaska USA; ^2^ National Oceanic and Atmospheric Administration, Alaska Fisheries Science Center, Resource Ecology and Fisheries Management Seattle Washington USA; ^3^ Museum of the North, University of Alaska Fairbanks Fairbanks Alaska USA

**Keywords:** Bering Sea, forage fish, Gulf of Alaska, low‐coverage whole genome resequencing, population genomics

## Abstract

Forage fishes are biological drivers throughout the Pacific Ocean, from the Arctic to nearly subtropical latitudes. As a critical trophic link, the health and stability of Pacific herring ( *Clupea pallasii* ) populations have implications for other marine species, including several targeted by large, productive fisheries. Previous research has indicated marked divergence between Pacific herring in the Bering Sea and the Gulf of Alaska. Seeking to localize this biogeographic break, we generated low‐coverage whole genome resequencing data for 120 Pacific herring from seven sites across the northern Gulf of Alaska and the eastern Bering Sea and Aleutian Islands. Single nucleotide polymorphisms across the mitogenome (267) and nuclear genome (~5.6 million) corroborate a biogeographic break in Pacific herring along the Alaska Peninsula and Aleutian Islands, as far west as Unalaska. We identified two distinct populations: one exists along the northern coasts of the Aleutian Islands and in the eastern Bering Sea; the other occupies the southern edge of the Aleutians and the Gulf of Alaska. Two mitochondrial haplogroups co‐occurring across the Gulf of Alaska suggest secondary contact between two populations, likely representing glacial refugia. Our results underscore the importance of geological events to contextualize the diversification of forage fish species.

## Graphical abstract

As a critical trophic link, the health and stability of Pacific herring ( *Clupea pallasii* ) populations have implications for other marine species, including several targeted by large, productive fisheries. We identified two distinct populations of Pacific herring in the North Pacific, mitonuclear discordance in the Gulf of Alaska, and cryptic diversity in the eastern Bering Sea. Our results underscore the importance of geological events to contextualize the diversification of forage fish species.

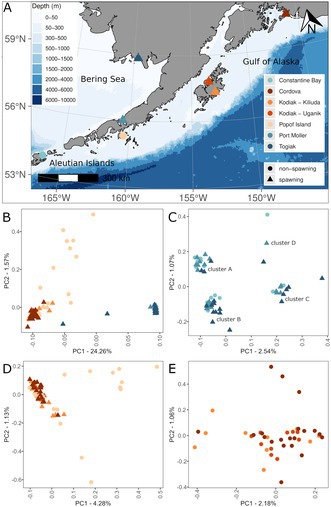

## 1 Introduction

Forage fishes are small‐ to medium‐sized species that function as linchpins of the trophic web, consuming algae and plankton and serving as abundant prey items for commercially important fishes, marine mammals, and seabirds (Surma, Pitcher, et al. 2018). Though taxonomically diverse, forage fishes are unified by their abundance and “boom‐and‐bust” population dynamics (Trochta et al. 2020). Globally, forage fishes are economically valuable and provide a variety of ecosystem services (Pikitch et al. 2014; Konar et al. 2019; Lam et al. 2019; Nissar et al. 2023). Several species also hold important roles in Indigenous cultures (Morin et al. 2023), including Pacific herring (Thornton 2015; Moss 2016), eulachon (Beveridge et al. 2020), and smelt (Palmer et al. 2018). Forage fishes are especially vulnerable to rising ocean temperatures. At high latitudes, where waters are warming 2×–4× faster than the global average (Chylek et al. 2022; Rantanen et al. 2022), higher sea temperatures are associated with detrimental effects on growth rate, body size, and nutritional value of forage fish species (Gobler et al. 2018; Hollowed et al. 2012; Robards et al. 2002; von Biela et al. 2019). Despite their ecological, economic, and cultural importance, and their vulnerability to abiotic factors associated with climate change, forage fishes are understudied in the Arctic, with a paucity of genetic data in particular (Timm et al. 2023).

Pacific herring ( *Clupea pallasii* ) is a key forage fish species driving biological processes throughout the North Pacific Ocean, from the Arctic to nearly subtropical latitudes (Hay and McCarter 1997). Due to Pacific herring's role as a link between trophic levels, its population health and stability have critical implications for other marine species: the high energy content of Pacific herring makes it a valuable prey species for marine mammals (Surma, Pitcher, et al. 2018; Surma, Pakhomov, and Pitcher 2018), seabirds (Bishop et al. 2015), and piscivorous fishes, including several targeted by large, productive fisheries (e.g., walleye pollock, Pacific salmons, Pacific halibut) (Brodeur et al. 2014). Annual Pacific herring spawning events deliver an influx of energy to nearshore environments that attract a taxonomically diverse array of predators, while also supporting herring egg subsistence fisheries (Thornton 2001, 2015; Surma et al. 2021). In Alaska, USA, Pacific herring fisheries are managed in geographically designated units and include subsistence, sac roe, and bait harvests (Woodby et al. 2005). The Northeast Pacific herring sac roe fishery has held high commercial value in Japanese markets since the 1970s (Lam et al. 2019). Sac roe harvest corresponds with spring spawning events, while the food and bait fisheries occur during the rest of the year, targeting non‐spawning adult herring and possibly targeting adults from multiple spawning populations, the largest of which is in Togiak, Alaska, USA.

Multiple methodologies have been used to characterize the demographic and population structure of Pacific herring in Alaskan waters. Herring in the Bering Sea are longer lived (16 years versus 8 years), mature later (3 to 5 years versus 2 years), and reach notably larger body sizes (asymptotic weight 520 g versus 297 g) than East Pacific populations (Hay et al. 2008). However, comparisons of other physical characteristics including growth and maturation rates, scale age patterns, fatty acid composition, and otolith microchemistry have been unable to reliably describe population structure within oceanic basins (Wespestad and Barton 1979; Rowell 1981; Otis et al. 2010).

**FIGURE 1:**
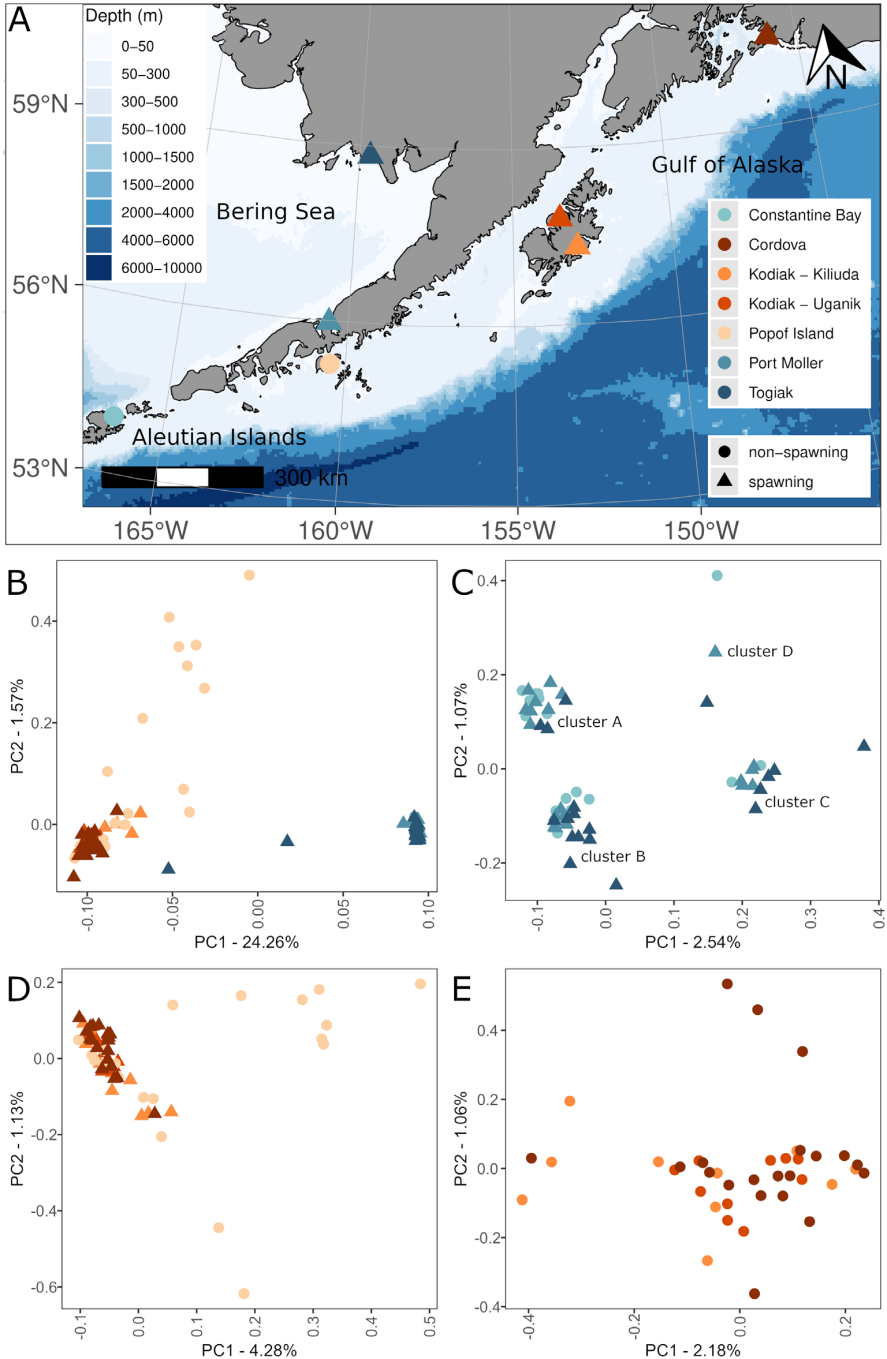
(A) Sampling locations of *Clupea pallasii* from the north Pacific Ocean and eastern Bering Sea. Symbol color indicates collection site and symbol shape indicates whether samples were obtained from spawning (▲) or non‐spawning (●) fish aggregations. PCA results from the full set of nuclear genome SNPs (B) for all samples; (C) samples from eBSAI (we denote the upper left cluster as A, the lower left cluster as B, the lower right cluster as C, and the upper right cluster as D); (D) samples from nGOA, including Popof Island; and (E) samples from nGOA, excluding Popof Island (all samples in this plot are from spawning aggregations).

Previous efforts to characterize the genetic structure of Pacific herring across their geographic range identified marker‐dependent patterns of variation. Allozyme variation indicated the presence of two Pacific herring populations: one occupying the eastern Gulf of Alaska and one inhabiting the Bering Sea and Northwest Pacific, with a genetic break along the Alaska Peninsula (Figure 1A; Grant and Utter 1984). Analysis of the mitochondrial cytochrome *b* gene (cytB) confirmed the Northwest Pacific/Bering Sea population and identified some intrusion of those haplotypes into the East Pacific, as far as Haida Gwaii off the coast of British Columbia (Liu et al. 2011). Additionally, two other mitochondrial lineages were found to co‐occur and dominate East Pacific populations (Liu et al. 2011). Results from the mitochondrial control region closely reflected cytB results, and while analysis of 10 microsatellite loci nearly recapitulated the allozyme signal, separation was made between the East Pacific, including Togiak in the eastern Bering Sea, and the Northwest Pacific, with some admixture between the two regions (Liu et al. 2012). The observed mitonuclear discordance was hypothesized to be an outcome of secondary contact between lineages that diverged in vicariance during the mid‐Pleistocene, approximately 1.3 mya, when glaciation mediated climate cooling would have driven the species into disjunct ranges (Liu et al. 2011, 2012). Microsatellites and mitochondrial markers did not yield evidence of significant genetic structure within the Bering Sea, but suggested a break within Prince William Sound, differentiating an eastern and western population in the Gulf of Alaska (O'Connell et al. 1998; Wildes et al. 2011, 2018). Within spawning seasons, observations of isolation‐by‐distance indicated geographical and seasonal spawn‐site fidelity, suggesting geographically limited gene flow (Petrou et al. 2021). Prior to the application of next generation sequencing, molecular investigations of Pacific herring predominantly showed regional differences that did not necessarily correspond with spawning stocks. However, a different demographic picture is now emerging.

Whole genome resequencing methods, specifically, are revolutionizing population genetics by addressing information gaps across the Tree of Life (Funk et al. 2012; Hemmer‐Hansen et al. 2014). “Big Data” enhances our ability to inventory adaptive variation (Nielsen et al. 2009), characterize genetic structure with high precision, and identify genomic features such as sex‐determining regions (Hansen et al. 2022) and structural variants (Mérot et al. 2020). Genotype likelihoods calculated from low‐coverage whole genome resequencing (lcWGS) datasets increase capacity for genome‐scale analysis of large sample sizes (Lou et al. 2021). During the past decade a suite of computational tools has emerged to analyze genotype likelihood datasets and, consequently, lcWGS has gained popularity for investigating genetic population structure in marine species (Clucas et al. 2019; Howe et al. 2024; Timm et al. 2024; St. John et al. 2025), including a variety of forage fish species (Andersson et al. 2024).

Here, we aim to improve our understanding of nuclear and mitochondrial population structure in Pacific herring across the large marine ecosystems in the Northeast Pacific. We used lcWGS to (1) characterize the genetic structure of Pacific herring spawning populations from Northeast Pacific sites spanning both previously hypothesized biogeographic breaks; (2) localize the biogeographic break between basins with increased precision; and (3) examine mitonuclear discordance in the species. Our results reveal stark examples of multifaceted cryptic diversity harbored in subarctic Pacific herring populations.

## 2 Methods

### 2.1 Sampling

We sampled 120 herring from seven collection sites in the northern Gulf of Alaska (nGOA) and eastern Bering Sea and Aleutian Islands (eBSAI) (Figure 1A; Tables 1, A1). Herring samples from Cordova, Togiak, Port Moller, and two locations around Kodiak Island were reproductively mature fish caught from active spawning aggregations. Herring samples from Constantine Bay and Popof Island were caught from non‐spawning aggregations outside of the spawning season. Samples were collected by state and federal agency biologists and were obtained from the Alaska Department of Fish and Game Gene Conservation Lab archive of tissue samples.

**TABLE 1:** Information about the geographic regions included in this study, including the collection regions, sample sizes (*N*), collection date ranges, and diversity values associated with the two‐population scenario, including nucleotide diversity (π) and heterozygosity (average individual SFS), and mitochondrial (mt) haplotype and nucleotide diversity.

Region	*N*	Collection date range	π	SFS	mt haplotype diversity	mt nucleotide diversity	Population	*π*	SFS	mt haplotype diversity	mt nucleotide diversity
**Cordova**	20	March 2023	0.323	0.311	1.000	0.167	nGOA	0.308	0.330	0.999	0.197
**Kodiak—Uganik**	10	April 2023	0.329	0.299	1.000	0.252					
**Kodiak—Kiliuda**	10	April 2023	0.329	0.308	1.000	0.184					
Popof Island	20	July 2023	0.334	0.375	1.000	0.265					
**Port Moller**	20	May 1996	0.320	0.270	0.963	0.137	eBSAI	0.300	0.273	0.943	0.136
Constantine Bay	20	July 1999	0.321	0.269	0.947	0.143					
**Togiak**	20	May 2022	0.310	0.282	0.900	0.142					

*Note:* Spawning aggregations are in bold.

### 2.2 Whole Genome Library Preparation and Sequencing

Genomic DNA was extracted from tissue samples using the Qiagen PureGene Tissue kit following the manufacturer's protocol (Qiagen, Maryland, USA). Genomic DNA was run on 1% agarose gels to assess quality. Genomic DNA was quantified with a Qubit 4.0 fluorometer using a dsDNA broad range assay kit (Thermo‐Fisher Scientific, USA). Library preparation and sequencing were conducted at the Oklahoma Medical Research Foundation NGS Core (Oklahoma City, OK, USA). Whole genome libraries were constructed using an xGen DNA EZ Library Prep Kit, paired with xGen Normalase UDI primers (Integrated DNA Technologies, Iowa, USA) and KAPA Pure Beads (Roche Molecular Systems, USA) in preparation for paired‐end sequencing of 150 bp. Samples from Togiak and Cordova were sequenced on an Illumina NovaSeq 6000 S4 at up to 6× coverage. Samples from remaining locations were sequenced on an Illumina NovaSeqX with up to 4× coverage.

### 2.3 Data Filtering, Alignment, and Calculation of Genotype Likelihoods

Quality of raw reads was ascertained with FastQC v0.12.0 (Andrews 2010) and aggregated with multiQC v1.19 (Ewels et al. 2016). The TRIMMOMATIC v0.39 (Brodeur et al. 2014) *ILLUMINACLIP* tool was used to identify and remove adapters and Illumina‐specific sequences (*2:30:10:1:true*), only retaining reads > 40 bp post‐trimming (*MINLEN:40*). We clipped polyG tails using *trim_poly_g* (‐*L ‐A –cut_right*), implemented in fastp v0.23.2 (Chen et al. 2018), to remove a known artifact of two‐dye sequencing chemistries.

The Atlantic herring ( *Clupea harengus* ) reference genome (GCF_900700415.2; Pettersson et al. 2019) was indexed with *index* in BWA v0.7.17 (Li and Durbin 2009) to facilitate alignment of raw reads to the reference genome. Following trimming and clipping, paired reads were aligned to the indexed reference genome with BWA's *mem* algorithm. We marked shorter split hits as secondary (*‐M*), to ensure alignment results were compatible with downstream filtering programs. Individual alignments were sorted by coordinates with the *sort* tool in samtools v1.19 (Danecek et al. 2021) before removing duplicate reads arising from PCR with the *MarkDuplicates* tool in Picard v2.26.6. Next, overlapping sequence ends were clipped for each mapped read pair with the bamutil v1.0.15 (Table A2) *clipOverlap* tool. Finally, we calculated sequencing depths across the genome of every individual using the *depth* tool in samtools and estimated the mean sequencing depth for each individual using a custom Python script (Table A2). All individuals had a mean sequencing depth > 1× and were included in genotype likelihood calculations (Lou et al. 2021).

Genotype likelihoods were estimated for single nucleotide polymorphisms (SNPs) with the samtools model (‐*GL 1*) as implemented in ANGSD v0.940 (Korneliussen et al. 2014). For a site to be considered polymorphic, the minimum and maximum depth thresholds were set to the number of individuals in the dataset (*‐setMinDepth 120*) and 5× the number of individuals in the dataset (*‐setMaxDepth 600*), respectively. Major and minor alleles were inferred from genotype likelihoods (*‐doMajorMinor 1*). Candidate SNPs were removed from downstream analysis when: sequencing quality or mapping quality scores were < 15 (*−minQ 15 ‐minMapQ 15*); the *p*‐value for polymorphism was greater than 10^−10^ (*‐SNP_pval 1e‐10*); and the minor allele frequency (MAF) was < 0.05 (*−minMaf 0.05*). Paralogous loci were identified by reading SNP‐wise coverage statistics from samtools' *mpileup* tool into ngsParalog v1.3.4 (Table A2). First, SNPs from reads marked as unmapped or duplicated were disregarded and base and mapping quality thresholds were explicitly set to 0 (*‐q 0 ‐Q 0 –ff UNMAP,DUP*). Mpileup results were piped directly to ngsParalog to calculate the likelihood ratio that each SNP was mismapped to a paralogous site. A *Χ* ^2^ test and Bonferroni correction were used to identify and exclude SNPs with adjusted *p*‐values > 0.05.

### 2.4 Inference of Population Genetic Structure With Nuclear Data

Genetic variability and structure across the whole dataset were examined with principal component (PCA) and admixture analysis on genome‐wide SNPs. We calculated a covariance matrix from SNP genotype likelihoods in PCAngsd v0.99 (Meisner and Albrechtsen 2018), using the minimum average partial test (*‐e*) to determine the number of eigenvalues to retain (Velicer 1976). We generated eigenvectors by decomposing the covariance matrix with *eigen*, as implemented in R v4.2.0 (R Core Team 2021). Results from PCA were first used to ascertain whether combining data from two sequencing runs introduced genetic structure artifacts (Lou et al. 2021; Lou and Therkildsen 2022). After estimating the position of each individual along PC1, we performed a linear regression analysis to estimate the correlation between PC1 position and sequencing depth. For admixture analysis, we tested *K* = 1–7 using three replicates in NGSadmix (Skotte et al. 2013) and the *K* with the highest log likelihood was deemed optimal.

### 2.5 Differentiation, Diversity, and the Identification of Genomic Outlier Regions

The Atlantic herring reference genome was indexed with *faidx* in samtools to facilitate site frequency spectra estimation in ANGSD. This reference genome was used as the ancestral genome (*‐anc* same as *‐ref*) in the folded site allele frequency likelihood (SAF) calculation for each region with *doSaf* in ANGSD. Parameters set for genotype likelihood calculation were used for SAF calculation, with depth thresholds tuned to reflect the sample size of each region. Regional SAFs were used to estimate the 2D site frequency spectrum (SFS) and pairwise *F*_*ST*_ with *realSFS* and *realSFS fst*, respectively, in ANGSD. SAFs were also used to calculate diversity and heterozygosity statistics for each collection region and each population using *realSFS saf2theta* and *thetaStat* in ANGSD.

Statistical significance of pairwise *F*_*ST*_ values was determined with an individual‐based permutation test, as implemented in Timm et al. (2024). Holding population number and sample sizes constant, every permutation randomly assigned individuals to populations, without replacement, and calculated weighted *F*_*ST*_. Fifty permutations were completed to generate a distribution of weighted *F*_*ST*_ values. Using a custom Python script (Table A2), the mean of this distribution was calculated and used to estimate the cumulative distribution function (CDF) of the *F*_*ST*_ values under an exponential distribution with *p*‐value = 1—CDF (Elhaik 2012). *p*‐values less than 0.05 were deemed statistically significant.

Pairwise *F*_*ST*_ was estimated between all collection sites and between regions (eBSAI and nGOA) to assess genome‐wide differentiation. Site allele frequency likelihoods were used to estimate 2D SFS and calculate *F*_*ST*_ by site (*realSFS fst stats2*). By‐site *F*_*ST*_ values were visualized in Manhattan plots. Spans of elevated *F*_*ST*_ were further investigated with a local score approach (Fariello et al. 2017; Andrews et al. 2023) prior to designation as an outlier region. For each group, SNPs were filtered by MAF (> 0.05), mapping quality (> 15), and depth (*n* ≤ depth ≤ *n* * 5, where *n* is the number of samples in the group) prior to counting alleles for each SNP in each group (*angsd ‐do Counts 1 ‐dumpCounts 3*). Targeting SNPs from both groups, local score analysis uses Fisher's Exact Test to identify SNPs with allele frequencies that differ significantly from the background allele frequency distribution for each group pair and analyze their proximity with a smoothing parameter (*ξ* = 2) (Fariello et al. 2017). Chromosome‐specific significance thresholds were calculated with *α* = 0.01 and genomic regions exceeding the corresponding significance threshold were designated as outlier regions. To qualify as a genomic region of interest, the loci needed to be (1) under selection (identified by local score analysis) and (2) differentiating between groups (contain SNPs with *F*_*ST*_ in the top 1% of values).

When a genomic region of interest was identified, variation was investigated further with a PCA of SNPs within the genomic region. We also constructed a genotype heatmap using a custom script that calculated a single estimate of allele dosage from the three genotype likelihoods associated with each locus for each individual (Table A2). Finally, linkage disequilibrium (LD) was calculated as the Pearson correlation coefficient (*r* ^2^) between all polymorphic sites within the genomic region with ngsLD v1.2.1 (Fox et al. 2019).

### 2.6 Inference of Population Genetic Structure With Mitogenomic Data

As mitochondrial SNPs had sufficient sequencing depth to confidently call genotypes (> 10×) and a species‐specific mitochondrial genome was available, mitogenomic haplotypes were analyzed separately from nuclear SNPs. Quality filtering and data assembly followed the process described above, though trimmed and clipped reads were aligned to the Pacific herring mitochondrial genome (NC_009578.1; Lavoue et al. 2007) and minimum and maximum sequencing depths were tuned to the mitochondrial alignment. Genotype likelihood data were converted to a fasta file with a custom Python script (Table A2), coding indeterminate genotype likelihoods (< 0.99) as missing data. We also removed any sites for which genotype likelihood was > 0.99 for the heterozygous state, as that is impossible in the haploid mitogenome. Quality checks were performed and individuals with > 10% missing data (*n* = 13) were removed from the alignment using Geneious Prime v2024.0.5.

Population genomic analysis of mitochondrial SNPs was performed in R. Haplotype and nucleotide diversity statistics were calculated for each collection region and for each population with *pegas* v1.3. PCA was conducted with *glPCA* from *adegenet* v2.1.5 (Table A2). PopART v1.7 (Leigh and Bryant 2015) was used to plot the minimum spanning haplotype network. We conducted an Analysis of Molecular Variance (AMOVA) with *poppr.amova* and calculated pairwise *F*_*ST*_ with *pairwise.WCfst* in *poppr* v2.9.3 (Table A2). Statistical significance was calculated with the individual permutation test implemented in *test.between* in *hierfstat* v0.5‐10, specifying 1000 permutations (Table A2).

### 2.7 Localizing the Biogeographic Break

We employed an isolation‐by‐distance (IBD) approach to localize the biogeographic break between the nGOA and eBSAI identified by previous studies, comparing genetic distance (*F*_*ST*_/(1—*F*_*ST*_)) to geographic distance (km) between collection sites, the latter of which was ascertained by plotting the shortest distance between collection sites that did not cross land. We assigned collection sites to groups previously identified by Liu et al. (2011, 2012). Data points were categorized by whether the corresponding pair of collection sites was in physical proximity and thus represented one group or two. For example, Constantine Bay and Port Moller are both located in the eBSAI, so the corresponding pairwise comparison was categorized as “within group,” whereas Togiak and Cordova are located within the eBSAI and nGOA, respectively, so this pairwise comparison was categorized as “between groups.”

To determine whether the groups described in previous research were also identifiable in whole genome data, we tested for a biogeographic break between the Northeast Pacific and the Bering Sea (Grant and Utter 1984; Liu et al. 2011) with a Wilcoxon‐Mann–Whitney test comparing *F*_*ST*_ values within groups versus between groups.

### 2.8 Mitonuclear Discordance

We categorized all fish by their mitochondrial haplogroup, calculated *F*_*ST*_ between these groups across nuclear sites and visualized results on Manhattan plots to identify discordant variation between nuclear and mitochondrial genomes. Finally, we used the local score approach described above to identify proximal SNPs whose allele frequencies significantly differed from the background distribution.

### 2.9 Computational Resources

All plots were generated and several analyses were conducted in R, using packages detailed and cited in Table A2. Figures were prepared for publication in GIMP (GNU Image Manipulation Program). Detailed scripts for data assembly, analysis, and visualization can be found at https://github.com/letimm/pacific‐herring_lcWGS.

## 3 Results

### 3.1 Sequencing and Genotype Likelihood Estimation

Six geographic regions were represented by 20 individuals each, totaling 120 samples (Tables 1, A1). Sequencing yielded 1,799,068,017 raw paired‐end sequences (approximately 14,992,233 sequences per individual). Raw reads were deposited in the National Center for Biotechnology Information's Sequence Read Archive under BioProject PRJNA1356517. All individual alignments had an estimated mean sequencing depth > 1×, with an overall mean of 3.22× (standard deviation = 0.54). Initially, 5,770,843 SNPs were genotyped across all individuals, but 180,993 SNPs (3.1%) were classified as paralogous and removed, resulting in a final set of 5,589,850 SNPs.

Mean sequencing depth estimated for alignment to the *Clupea pallasii* mitogenome was 412.35× (s.d. = 347.40) across 120 individuals. After removing 10 paralogous SNPs, 267 SNPs were retained.

### 3.2 Inference of Population Genetic Structure With Whole Genome Data

**FIGURE A1:**
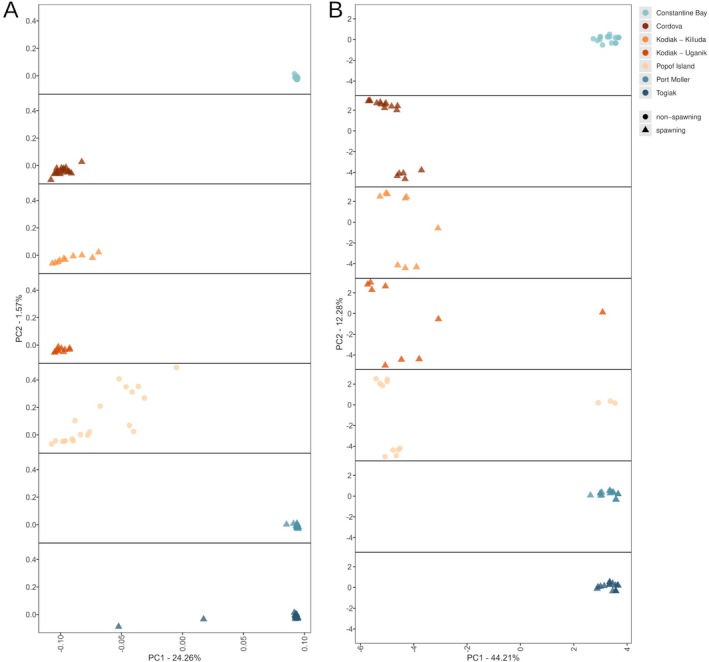
PCAs by collection region for (A) nuclear and (B) mitogenomic data. Color indicates collection region and shape reflects spawning status.

Principal component analysis (PCA) revealed two primary clusters differentiated along PC1 (24.26% variance) that corroborated the split between the Gulf of Alaska and Bering Sea: one cluster contained individuals from Cordova, Kodiak‐Kiliuda, Kodiak‐Uganik, and Popof Island (the nGOA population) and the other cluster contained samples from Constantine Bay, Togiak and Port Moller (the eBSAI population) (Figures 1B, A1A). Two individuals from Togiak fell between the eBSAI and nGOA clusters. Aside from these individuals, the eBSAI cluster was more tightly grouped; the nGOA cluster exhibited greater spread and, while it contained 10 individuals from Popof Island, half of the individuals from Popof Island spread along PC2 (1.57% variance). Given the high variance explained along PC1, hierarchical PCA was employed to better visualize individuals in PC‐space. Individuals in the eBSAI and nGOA clusters were re‐analyzed separately with PCA (both including and excluding Popof Island samples). While the eBSAI PCA identified four primary clusters, these clusters did not correspond to collection sites: all three eBSAI collection sites were present in every cluster (Figure 1C). When Popof Island was included in the nGOA PCA, differences between Popof Island and the other sites drove clustering, separating along PC1 (4.28% variance) and PC2 (1.13% variance) (Figure 1D). When Popof Island was removed, no structure was discernible in the remaining nGOA samples and variance along PC1 and PC2 axes decreased (2.18% and 1.06%, respectively) (Figure 1E).

**FIGURE 2:**
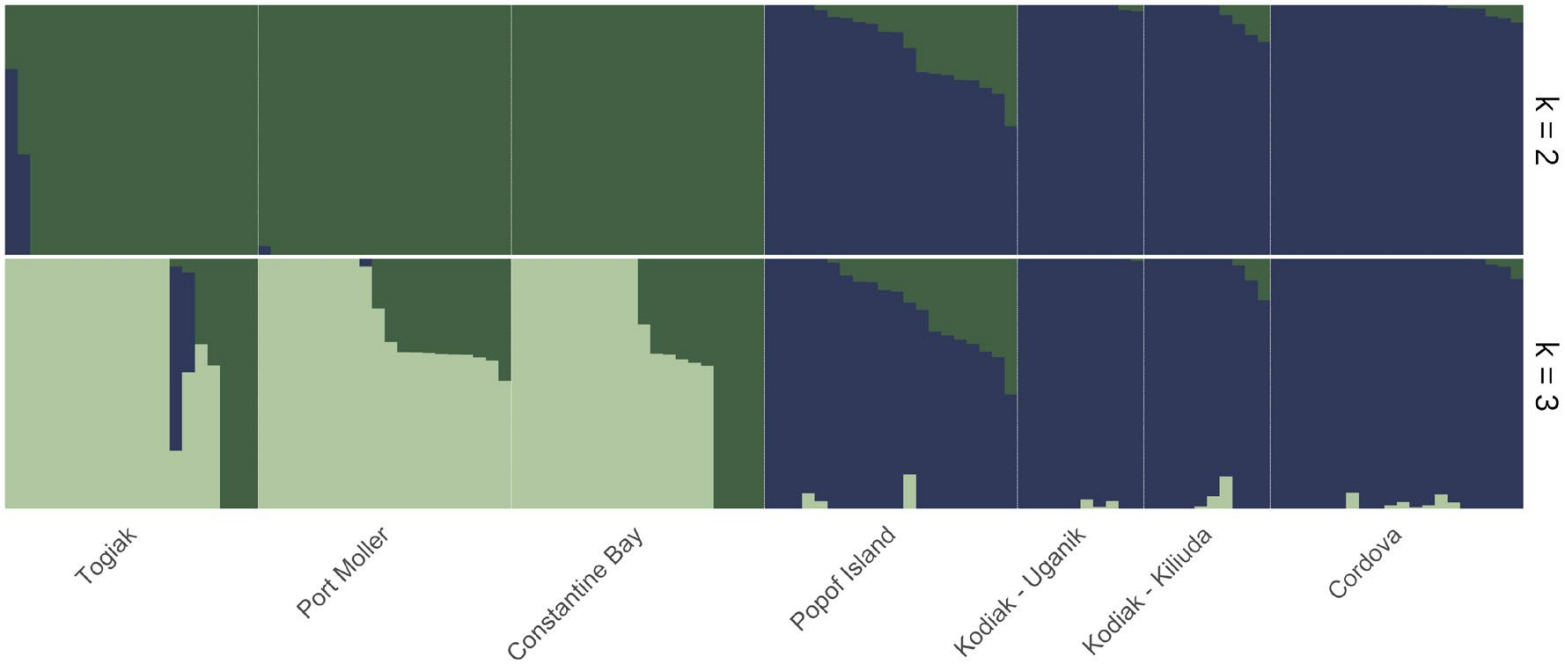
Ancestry proportions inferred by ADMIXTURE for *K* = 2 (log likelihood = ‘Inf’) and *K* = 3 (log likelihood = 484.38). Log likelihood was highest for *K* = 2, indicating optimality.

The results of individual admixture proportion analysis largely agreed with the results of hierarchical PCA (Figure 2). Testing *K* = 2 to *K* = 7 yielded log likelihood values ranging from 484.38 (*K* = 3) to infinite (*K* = 2, optimal). When *K* = 2, the groups recapitulated clusters identified with PCA: the eBSAI group contained all individuals from Constantine Bay, Togiak and Port Moller, with no admixture from the nGOA group (with the exception of two Togiak individuals which, in the PCA, appeared midway between the eBSAI cluster and the nGOA cluster). Similarly, the nGOA group contained all individuals from Cordova, Kodiak‐Kiliuda, Kodiak‐Uganik, and Popof Island with the greatest admixture with the eBSAI group estimated in individuals from Popof Island, a non‐spawning collection site. As *K* increased, we did not see further separation of discrete groups associated with collection sites. Rather, additional populations contributed proportions of admixture to eBSAI or nGOA. At *K* = 3, for instance, eBSAI individuals showed variable admixture proportions attributable to two populations.

### 3.3 Differentiation, Diversity, and the Identification of Genomic Outlier Regions

Pairwise *F*_*ST*_ values were calculated between eBSAI and nGOA, with Popof Island included in nGOA (two‐population scenario) or defined as a separate population (three‐population scenario), as well as between all pairs of collection sites (Table 2). Under the two‐population scenario, pairwise *F*_*ST*_ between eBSAI and nGOA was 0.178 (*p* < 0.001). This value increased when Popof Island was denoted as a unique population under the three‐population scenario (*F*_*ST*_ = 0.193; *p* < 0.001; Table A3). Pairwise *F*_*ST*_ was high and significant between Popof Island and eBSAI (*F*_*ST*_ = 0.151; *p* < 0.001), however, pairwise *F*_*ST*_ was low and insignificant between Popof Island and nGOA (*F*_*ST*_ = 0.022; *p* > 0.1). Because of this, subsequent analyses focused on the two‐population scenario: nucleotide diversity (π) was 0.300 in eBSAI and 0.308 in nGOA. Heterozygosity in eBSAI was 0.273 and 0.330 in nGOA. By collection region, π was highest in Popof Island (0.334) and lowest in Togiak (0.310). Popof Island also had the highest heterozygosity (0.375) and Constantine Bay had the lowest (0.269) (Table 1).

**TABLE 2:** Pairwise weighted *F*_*ST*_ values and associated *p*‐values are reported above and below the shaded diagonal, respectively. Note that collection sites are grouped by region, eBSAI and nGOA.

	Togiak	Port Moller	Constantine Bay	Popof Island	Kodiak—Uganik	Kodiak—Kiliuda	Cordova
Togiak		0.007	0.007	0.136	0.160	0.156	0.181
Port Moller	> 0.999		0.006	0.128	0.146	0.144	0.169
Constantine Bay	> 0.999	> 0.999		0.126	0.142	0.140	0.167
Popof Island	< 0.001	< 0.001	< 0.001		0.017	0.014	0.021
Kodiak—Uganik	< 0.001	< 0.001	< 0.001	0.511		0.006	0.010
Kodiak—Kiliuda	< 0.001	< 0.001	< 0.001	0.918	> 0.999		0.011
Cordova	< 0.001	< 0.001	< 0.001	0.171	> 0.999	> 0.999	

Pairwise *F*_*ST*_ values between collection sites within regions ranged from 0.006 (Kodiak‐Uganik versus Kodiak‐Kiliuda within nGOA; Constantine Bay versus Port Moller within eBSAI) to 0.021 (Cordova versus Popof Island within nGOA). Intra‐population *F*_*ST*_ values were not statistically significant (Table 2). Between all combinations of collection sites across regions, pairwise *F*_*ST*_ ranged from 0.126 (Constantine Bay versus Popof Island) to 0.181 (Cordova versus Togiak) and all inter‐population comparisons were statistically significant (*p* < 0.001) (Table 2).

**FIGURE 3:**
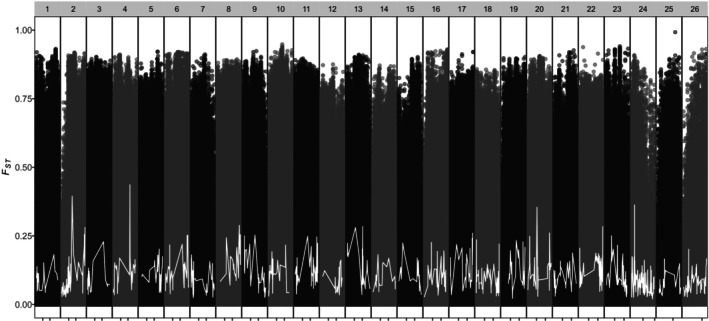
Manhattan plot of *F*_*ST*_ values for all SNPs comparing populations under the two‐population scenario: NGOA (Cordova, Kodiak, and Popof Island) vs. eBSAI (Togiak, Constantine Bay, and Port Moller). The sliding window average *F*_*ST*_ is denoted by a white line.

Genome scans of *F*_*ST*_ comparing populations under the two‐population and three‐population scenarios enabled us to query the data for adaptive loci at unprecedented resolution. However, the differentiation between eBSAI and nGOA was sufficiently high in both scenarios to preclude the identification of adaptive regions (Figure 3).

**FIGURE A2:**
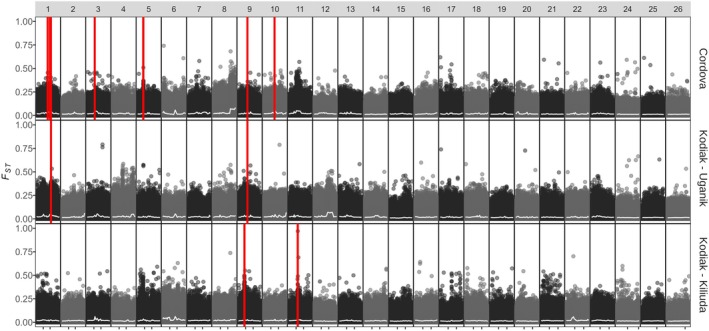
Manhattan plot of *F*_*ST*_ values for all SNPs comparing Popof Island vs. Cordova (top), Kodiak—Uganik (middle), and Kodiak—Kiliuda (bottom). Vertical red lines indicate genomic regions identified as under selection by local score analysis. The sliding window average *F*_*ST*_ is denoted by a white line.

**FIGURE A3:**
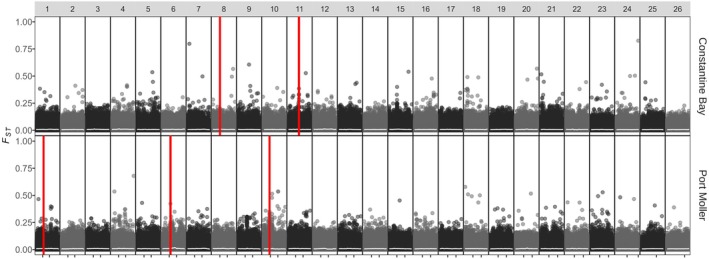
Manhattan plot of *F*_*ST*_ values for all SNPs comparing Togiak vs. Constantine Bay (top) and Port Moller (bottom). Vertical red lines indicate genomic regions identified as under selection by local score analysis. The sliding window average *F*_*ST*_ is denoted by a white line.

Comparisons between Popof Island and the remaining nGOA sites revealed several genomic regions of elevated *F*_*ST*_ (Figure A2), which were further investigated with the local score approach. Across all pairwise comparisons between collection sites within the nGOA, we identified a cumulative 19 genomic regions as potentially under selection. Most pairwise comparisons only identified 2–3 genomic regions, rarely on the same chromosome. The Cordova‐Popof Island comparison, however, identified seven genomic regions putatively under selection, three of which occur on chromosome 1. Interestingly, the high *F*_*ST*_ block on chromosome 8 was not classified by local score (Figure A2). One SNP with high *F*_*ST*_ (0.970) fell within a genomic region identified by local score analysis on chromosome 11 when comparing Popof Island to Kodiak‐Kiliuda, but appears to stand alone. In contrast, pairwise comparisons between collection sites in eBSAI only identified five regions with elevated *F*_*ST*_: two between Constantine Bay and Togiak and three between Port Moller and Togiak, none of which co‐occurred on a chromosome (Figure A3). No genomic regions identified by local score in any of our comparisons included SNPs in the top 1% of *F*_*ST*_ values.

**FIGURE 4:**
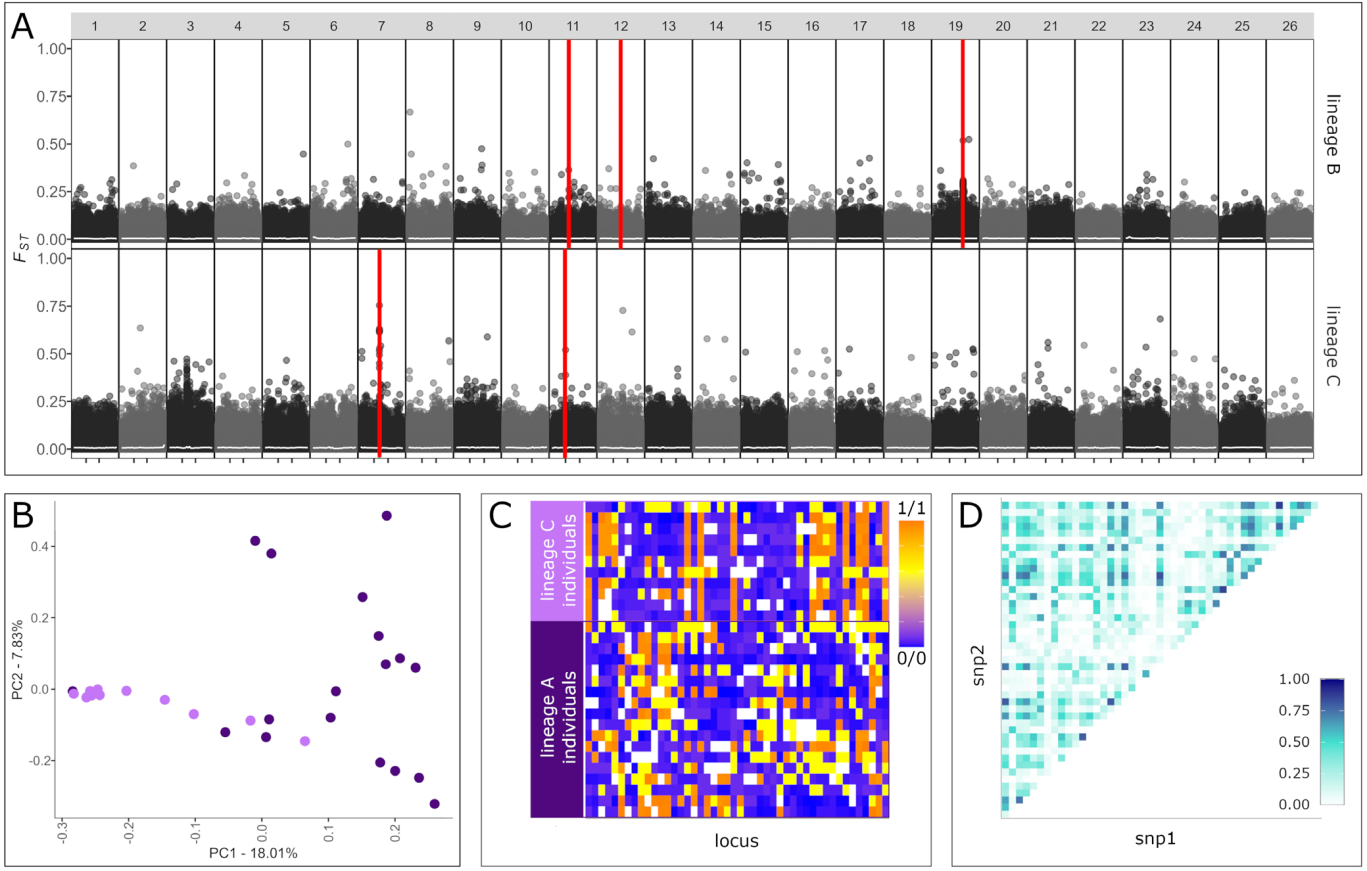
(A) Manhattan plot of *F*_*ST*_ values for all SNPs comparing eBSAI lineage A vs. lineage B (top) and lineage C (bottom). Vertical red lines indicate the regions identified as under selection by local score analysis. The sliding window average *F*_*ST*_ is denoted by a white line. Note the region on chromosome 7, where *F*_*ST*_ and local score identify *tmem8b*. This genomic region was further investigated with (B) a PCA of the 51 SNPs comprising the genomic region; (C) a genotype heatmap illustrating the likely allele dosage for each SNP (blue = likelihood of homozygosity for the major allele, 0/0, approaches 1; yellow = likelihood of heterozygosity approaches 1; orange = homozygosity for the minor allele, 1/1, approaches 1; intermediate colors reflect where heterozygosity and homozygosity both have likelihoods > 0; white = missing data or three equivalent genotype likelihoods); (D) a heatmap of LD, expressed as Pearson correlation coefficients (*r* ^*2*^), between all SNP‐pairs within the genomic region, where darker blue indicates higher LD.

Within the eBSAI, four clusters, denoted A–D, were identified in the PCA (Figure 1C), three of which had sufficient sample sizes to perform a genome scan of *F*_*ST*_ and local score analysis (Figure 4A). Local score analysis identified five genomic regions, two between lineages A and C and three between A and B. One genomic region identified by local score also contained SNPs with elevated *F*_*ST*_ (13 SNPs > 0.25; 8 SNPs > 0.50; 1 SNP > 0.75) on chromosome 7 in the A–C comparison (Figure 4A). NCBI's Genome Viewer indicated this region, spanning positions 13,841,600–13,851,100, fell within *tmem8b* (transmembrane protein 8B). PCA of the 51 SNPs between individuals from A and C within this genomic region generally separated the two lineages along PC1 (18.01%), though there was some overlap (Figure 4B). Individuals from lineage A were further distributed along PC2 (7.83%) while individuals from lineage C were not. The genotype heatmap revealed several loci with differences in allelic dosage between lineages A and C, though these were mostly individual SNPs and not blocks of adjacent sites (Figure 4C). Across 1036 SNP‐pairs within this genomic region, the average distance between SNPs was 3030 bp. Maximum *r* ^*2*^ = 0.874, average *r* ^*2*^ = 0.177, and *r* ^*2*^ ≥ 0.5 for 107 SNP‐pairs (Figure 4D).

### 3.4 Inference of Population Genetic Structure With Mitogenomic Data

**FIGURE 5:**
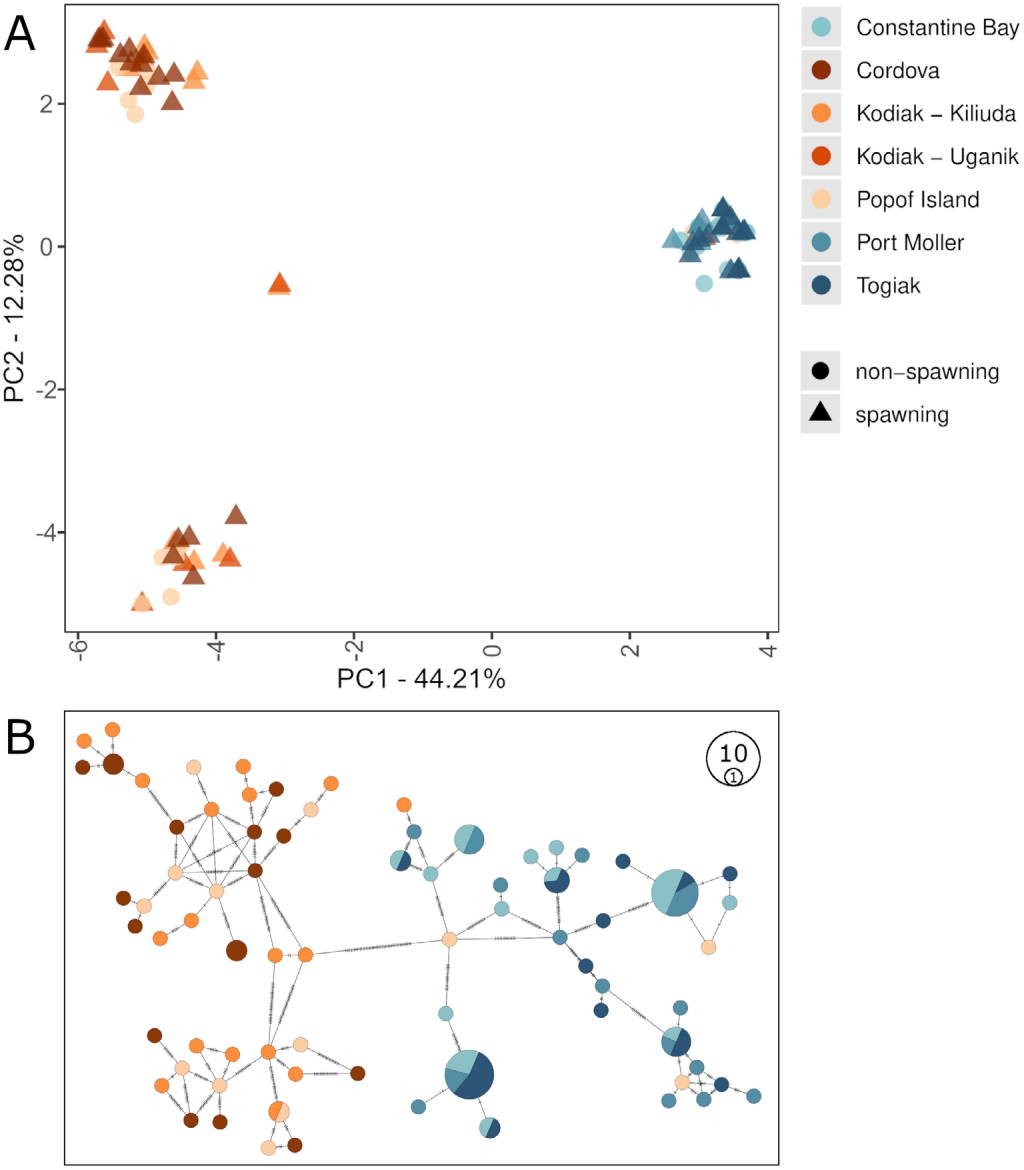
(A) PCA of mitochondrial haplotypes (267 variant sites) from all samples. (B) minimum spanning network of mitochondrial haplotypes. Node size indicates haplotype frequency and color indicates collection region. Mutations separating haplotypes are reflected as hatches.

Across the 16,700 bp mitogenome, 267 variant sites were identified across 107 individuals, representing 74 haplotypes. The PCA of these data revealed four haplogroups (Figure 5; Figure A1B): one was characteristic of the eBSAI (though one of these appears in Kodiak‐Uganik and three appear in Popof Island) and three were recovered only in fish from nGOA, where two haplogroups (GOA1 and GOA2) are dominant. An AMOVA found the majority of variance in the data (69.28%) could be attributed to differences between haplogroups and the remaining variance was exclusively associated with differences between individuals within collection regions. Differences between collection regions within each haplogroup contributed no variance. All estimates of pairwise *F*_*ST*_ were statistically significant and ranged from 0.697 (eBSAI versus GOA1; eBSAI versus GOA2 = 0.694) to 0.654 (GOA1 vs. GOA2). These results agreed with the haplotype analysis: eBSAI had 36 unique haplotypes, seven of which were shared between as many as 11 individuals across three collection sites (Constantine Bay, Port Moller, and Togiak). GOA1 had 15 unique haplotypes, two of which were shared between two individuals and only one of these spanned collection regions (this haplotype was found in Kodiak‐Uganik and Popof Island). GOA2 had 26 unique haplotypes, with only one shared between two individuals in Cordova (Figure 5).

The nGOA had higher mitochondrial haplotype (0.999) and nucleotide diversity (0.197) estimates than eBSAI (0.943 and 0.136, respectively) (Table 1). All collection sites in the nGOA population had haplotype diversity equal to one, compared to those in eBSAI, which ranged from 0.900 in Togiak to 0.963 in Port Moller. Mitochondrial nucleotide diversity was also higher among collection sites in the nGOA, ranging from 0.167 in Cordova to 0.265 in Popof Island, compared to collection sites in the eBSAI, which fell between 0.137 in Port Moller and 0.143 in Constantine Bay (Table 1).

### 3.5 Localizing the Biogeographic Break

**FIGURE A4:**
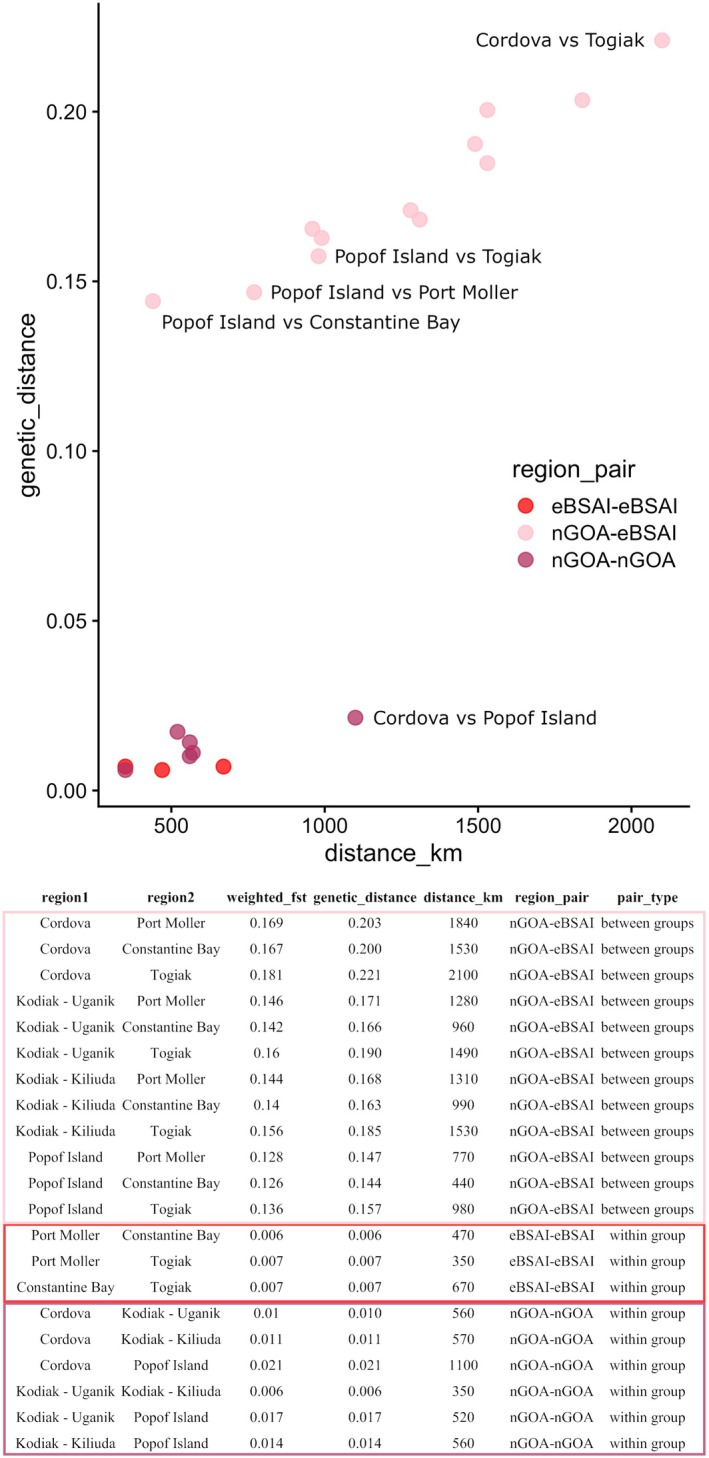
Top: Plot of genetic distance (*F*_*ST*_/(1—*F*_*ST*_)) by geographic distance between all collection region pairs, categorized as within eBSAI (red), within nGOA (maroon), or between collection regions representing eBSAI and nGOA (pink). Bottom: Table of the plotted values is provided.

Analysis of IBD using nuclear SNP data revealed a strong contrast in genetic distances between collection sites corresponding to the “within group” and “between groups” categories (Figure A4). Comparing genetic distance to geographic distance revealed two distinct categories: “within group” genetic distances (comparing two collection sites within nGOA or within eBSAI) were lower than “between group” genetic distances (comparing collection sites between nGOA and eBSAI). Notably, low geographic distance did not correspond to low genetic distance when sites were “between groups”. For example, the geographic distance between Constantine Bay and Port Moller (within group) is nearly equivalent to the geographic distance between Constantine Bay and Popof Island (between group), but the genetic distance of the latter is 24 times greater: 0.006 and 0.144, respectively. When the “within group” data was further divided into “within nGOA” and “within eBSAI,” there were insufficient data points to test for IBD within each region.

Both nuclear and mitochondrial analyses identified a sharp boundary between the eBSAI and nGOA populations within the 400 km between Popof Island and Constantine Bay (Figure A1). No nGOA mitochondrial haplotypes were recovered in the eBSAI and only a few individuals sampled in the nGOA carried eBSAI mitochondrial haplotypes (Figure 5). The nuclear genome showed a broadly similar pattern, with the exception of two individuals sampled in Togiak that showed strong evidence of admixture between eBSAI and nGOA nuclear genomes (Figures 1B and 2).

As our data were not normally distributed, we compared distributions of *F*_*ST*_ values between categories with the Wilcoxon‐Mann–Whitney Test. The result of this test indicated significantly different distributions (*p*‐value = 0.0001), with *F*_*ST*_ values significantly higher in the “between groups” category.

### 3.6 Mitonuclear Discordance

**FIGURE A5:**
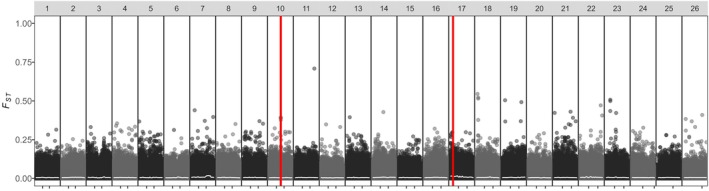
Manhattan plot of *F*_*ST*_ values for all nuclear SNPs comparing GOA1 vs. GOA2, as identified by mitochondrial haplotype. Vertical red lines indicate genomic regions identified as under selection by local score analysis. The sliding window average *F*_*ST*_ is denoted by a white line.

To investigate the degree of concordance between nuclear and mitochondrial data, we performed a genome scan of *F*_*ST*_ values and local score analysis comparing GOA1 and GOA2 in nuclear data (Figure A5). While a few SNPs had *F*_*ST*_ values > 0.5, none fell within the two regions identified by local score analysis: one on chromosome 10 and another on chromosome 17.

## 4 Discussion

We identify strong genetic diversification within Pacific herring following the divergence of herring into Atlantic and Pacific lineages facilitated by glacial melting and high sea levels (Laakkonen et al. 2013; Martinez Barrio et al. 2016; Jamsandekar et al. 2024). Our results provide the most extensive genetic evidence yet for two highly diverged populations of herring in the northeast Pacific. Nuclear and mitochondrial data corroborate a sharp biogeographic break between Bering Sea and northern Pacific populations north and south of the Alaska Peninsula and Aleutian Islands. Additionally, we identify at least three differentiated lineages of herring in the eBSAI that do not correspond to geographically isolated spawning populations. Finally, we confirm the existence of two distinct mitochondrial haplogroups co‐occurring in the northern Gulf of Alaska, likely an outcome of range shifts associated with changes in habitat availability resulting from global climate cycles.

### 4.1 The Alaska Peninsula and Aleutian Islands as a Biogeographic Break

Previously, the hypothesized location of a biogeographic break depended on the genetic marker analyzed: allozyme and mitochondrial data inferred a break along the Alaska Peninsula and Aleutian Islands (Grant and Utter 1984; Liu et al. 2011). Microsatellite variation inferred a break in the North Pacific, differentiating east from west (Liu et al. 2012). Our results concur with the former: we identified a population north of the Aleutian Islands and throughout the eastern Bering Sea and a population south of the Aleutian Islands and across the northern Gulf of Alaska.

The eBSAI haplogroup identified in our analyses corresponds to the mitochondrial lineage designated “A” by Liu et al. (2011), GOA1 and GOA2, represent lineages “B” and “C,” respectively, in Liu et al. (2011). In addition, two individuals sampled from Kodiak have mitochondrial genomes belonging to a fourth lineage present in public datasets, but not formally given a designation in any prior analysis of mtDNA variation (Liu et al. 2011). A phylogenetic analysis of whole mitochondrial genome sequences representing each of the four distinct haplogroups in our sample and rooted with a sequence from *C. harengus* supports a sister group relationship between the eBSAI lineage and a clade grouping the three nGOA haplogroups (unpublished data). Haplogroups from the nGOA clade are dominant in all examined populations from the northeast Pacific and are completely absent in the Bering Sea.

Our results also indicate that genetic differentiation between the eBSAI and nGOA populations is not limited to a specific genomic region but is genome‐wide (Figure 3). The margins of this division are clearest between Port Moller and Popof Island, two collection sites that are geographically near and exist on the same longitude. The spawning aggregation sampled from Port Moller groups strongly with the other eBSAI collection sites, while Popof Island shows a signal that corresponds to the nGOA population. Notably, several individuals from Popof Island cannot confidently be described as belonging to the nGOA or the eBSAI. Samples from Popof Island were not spawning, so these individuals may represent itinerant herring passing through the Aleutian Islands.

The genetic boundary we delineate does not appear to be impermeable: although we did not test for hybridization directly, we identified two individuals that fell between the eBSAI and nGOA clusters in the PCA and are indicated as admixed (Figures 1B and 2, A1A). It is notable that these two fish were collected from a spawning aggregation in Togiak; False Pass—the nearest point of entry to the Bering Sea from the nGOA—is approximately 600 km away. These admixed individuals carry eBSAI mitochondrial haplotypes, suggesting male‐driven introgression from the nGOA to the eBSAI. Elsewhere in the Bering Sea, individuals from Constantine Bay, which were all sampled outside the spawning season, exhibited no evidence of admixture, while a single fish from Port Moller showed a minor contribution of nGOA in a predominantly eBSAI nuclear genomic background. In contrast, introgression of eBSAI into nGOA is strongly indicated in the majority of individuals collected in Popof Island and, to a lesser extent in fish from other North Pacific collections (Figure 2).

Pacific herring exhibit a latitudinal cline, spawning earlier in the year at lower latitudes and later in the year at higher latitudes (Haegele and Schweigert 1985), presumably minimizing hybridization through reproductive allochrony. Spawn timing of Pacific herring is also regulated by annual temperature regimes (Hay 1985) and seasonality (Petrou et al. 2021), and genetic divergence associated with spawn timing has been identified as a driver of population structure in both Atlantic and Pacific herring (Lamichhaney et al. 2017; Petrou et al. 2021). However, spawning patterns in Pacific herring (Ljungström et al. 2019) and Atlantic herring (Winters and Wheeler 1996) are notably dynamic, even amongst geographically proximal populations. Herring spawn events have been documented in the nGOA from February to May, with spawning occurring later in the year as latitude increases (Rounsefell 1930); herring spawning has been recorded in the Bering Sea from late April to late May (Barton & Wepestad, 1980). This overlap in spawn timing, in concert with male herring developing earlier than female herring (Hay 1985), could explain the movement of male herring from the nGOA population into eBSAI spawning aggregations and resultant admixture between the populations.

The mitogenomic data indicated unidirectional straying in the opposite direction, from the eBSAI to the nGOA: four individuals from the eBSAI haplogroup were collected from two nGOA sites (Figure A1B). Three of these individuals were collected from Popof Island. This is unsurprising, given the close geographic proximity to Port Moller, an eBSAI spawning population, as well as the non‐spawning status of the Popof Island collection. Samples collected from Popof Island may have been passing through the region, rather than returning to spawn. The fourth stray, however, was collected between Kodiak Island and the Alaska Peninsula. This individual had a mitogenomic signal corresponding to the eBSAI, but clustered with nGOA in the nuclear PCA. This was surprising, given that the Alaskan Stream, the predominant oceanographic feature of the region, flows westward (Favorite 1967), penetrating to the benthos (Warren and Owens 1985). However, an eastward jet has been described flowing in the deep water south of the Aleutian Islands, north of the Alaskan Stream (Reed 1969, 1970; Warren and Owens 1988; Owens and Warren 2001), which may explain how an individual from the eBSAI haplogroup strayed so far into the nGOA.

Our results indicate this biogeographic break maintains the genetic distinctiveness of populations despite some permeability of individuals. Greater unidirectional flow exhibited in mitogenomic data suggests females from the eBSAI may disperse into the nGOA more frequently or with greater success than males and may reflect a general tendency for female herring to move further than males. Pacific herring are hypothesized to exhibit seasonal migrations to spawn and feed in deeper waters of the central Bering Sea, the patterns of which can be highly variable and dependent on environmental conditions such as sea‐ice extent and sea surface temperature (Tojo et al. 2007). Dispersal patterns of Pacific herring have been characterized to some degree, but not by sex. However, female herring mature slightly later than males (Hay 1985) and may use the additional maturation time to migrate further.

### 4.2 Adaptation and Cryptic Diversity With Gene Flow in the Eastern Bering Sea

Analyses of genetic differentiation suggest high levels of gene flow between spawning populations within the eBSAI and nGOA. However, we identified at least three sympatric lineages within the eBSAI, suggesting cryptic diversity in this region. Only one genomic region was identified as differentiating between lineages and as being under selection: this gene region corresponded to *tmem8b*, syn. nasopharyngeal carcinoma‐associated gene 6 (*ngx6*).

In humans, TMEM8B/NGX6 has been identified as a tumor suppressor (Yang 2000) and its downregulation is associated with several cancers (Zhang et al. 2003; Ma et al. 2005; Su et al. 2010; Lin et al. 2012). When functional, *ngx6* modulates several cell‐signaling pathways, including those associated with physiological processes of metabolism and mitosis (MAPK), intracellular signaling (PI3K/AKT), and proinflammatory signaling (NF‐κB) (Li et al. 2012). Two isoforms have been described and both share an epidermal growth factor (EGF)‐like extracellular domain. TMEM8B‐a is 134 amino acids longer than TMEM8B‐b and contains seven transmembrane domains compared to TMEM8B‐b's two domains (Wang et al. 2010). High expression of the TMEM8B‐b isoform is hypothesized to improve cell adhesion and reduce the capacity to invade other tissues (Peng et al. 2007).

In domesticated sheep, the only non‐human species in which *tmem8b* has been studied to date, *tmem8b* was identified as having been under extreme selection pressure (Kijas et al. 2012). Recently, Cinar et al. (2020) associated *tmem8b* genotype with mature weight at 3–4 years. across several sheep breeds, indicating selection for increased growth rate and larger body size during domestication drove selection at a SNP in *tmem8b*. Interestingly, Pacific herring in the Bering Sea are significantly larger than those in the Gulf of Alaska (Hay et al. 2008), and Bering Sea herring reach larger body sizes at higher latitudes (Schweigert et al. 2002). Hay et al. (2008) suggested this phenotypic difference was the result of local adaptation to resource availability and oceanographic regimes.

Detailed analysis of this genomic region does not indicate structural variation: the PCA does not cleanly separate lineages A and C (Figure 4B); the genotype heatmap indicates allelic dosages differ between lineages at several individual SNPs, rather than across blocks of adjacent variant sites (Figure 4C); and the LD heatmap does not indicate strong linkage between adjacent SNPs (Figure 4D). However, a high density of SNPs with elevated *F*_*ST*_ within a locus identified as experiencing selection suggests adaptive diversity. Because our results are from a single time point, it is not clear whether this adaptive signal is intensifying, being maintained, or diminishing. Each outcome gives rise to additional questions about the evolution of Pacific herring. If the signal is intensifying or maintained, what genomic mechanisms are facilitating adaptation with gene flow? If the signal is diminishing, what is preventing it from homogenizing on the timeline we expect (a few generations)? Answers to either question are critical to recapitulating the evolutionary history of high latitude Pacific herring.

### 4.3 Secondary Contact Between Glacial Refugia

Molecular clocks estimate the divergence between Atlantic and Pacific herring to have occurred during the Pliocene, approximately 1.5–4.2 mya (Grant 1986; Martinez Barrio et al. 2016; Wang et al. 2022; Jamsandekar et al. 2024). Northern GOA herring are hypothesized to have diverged from eBSAI herring during the Pleistocene, before further separating into two glacial refugia populations in the eastern Pacific approximately 1.0–1.3 mya (Morley and Dworetzky 1991; Liu et al. 2011). Assuming mitochondrial genome mutation rates are around 1.6% per million years (Burridge et al. 2008), we would expect to observe 266 SNPs between eBSAI and nGOA: we identified 267. During major cooling events in the Pleistocene, herring ranges likely shifted south where the three nGOA haplogroups diverged. Herring spawning habitat in the nGOA is estimated to have become increasingly available approximately 18,000 years ago (Hughes and Gibbard 2015) following the LGM, allowing for a northward shift of herring range and mixing of nGOA haplogroups.

Post‐glacial contact has been identified as a driver of current genetic population structure in a number of marine fishes in the North Pacific, including Pacific cod (Grant et al. 1987; Canino, Spies, Cunningham, et al. 2010), walleye pollock (Grant et al. 2010), and Atka mackerel (Canino, Spies, Lowe, and Grant 2010). In Pacific cod and walleye pollock, a secondary contact zone was identified in the North Pacific, separating Asian and North American populations, as initially reported for Pacific herring by Liu et al. (2012). Notably, Atka mackerel, another forage fish species, also reflected mitonuclear discordance, though trends differed from those described in Pacific herring: Canino, Spies, Lowe, and Grant (2010) report genetic homogeneity in Atka mackerel spanning Japan to the western Gulf of Alaska on the basis of nuclear and mitochondrial markers (microsatellites and the control region). However, mitochondrial diversity was much lower than expected, with only three haplotypes recorded from nearly 120 individuals, suggesting a population bottleneck or founder event associated with climate‐driven distribution shifts (Canino, Spies, Lowe, and Grant 2010).

The results of our study reify the importance of considering geological events when seeking to calibrate and understand patterns of species diversification observed in the present. Taken together, results from Atka mackerel and Pacific herring suggest that range shifts associated with global climate cycles must be considered when interpreting present‐day genetic population structure and mitonuclear discordance. Efforts to understand the complex interactions between environment and evolution in Pacific herring and other forage fish species may also prove informative for predicting the impact of future climate fluctuations on these key trophic links.

## 5 Conclusion

We identify two highly differentiated populations of Pacific herring: one occupies the eastern Bering Sea north of the Aleutian Islands and the other exists in the northern Gulf of Alaska, south of the Aleutian Islands. The lack of gene flow between these two populations of Pacific herring is similar to that described between Pacific and Atlantic herring, suggesting nGOA and eBSAI herring may represent distinct species. We hypothesize nGOA herring separated from eBSAI herring during the Pleistocene, but phylogenetic analysis with a molecular clock is necessary to estimate divergence timing. The lack of eBSAI–nGOA admixture is somewhat surprising given that Atlantic–Pacific hybrid herring are known to exist in stable populations in the Barents Sea (Pettersson et al. 2023) and Rossfjordvatn Lake in Norway (Strelkov et al. 2025). The lack of hybridization in the Pacific may be the result of reproductive allochrony, ocean current mediated segregation, or asymmetric reproductive incompatibility. Mitonuclear discordance in the nGOA underscores the importance of contextualizing population genetics in terms of the geological history of the region.

## Author Contributions

**Laura E. Timm:** data curation (lead), formal analysis (lead), visualization (lead), writing – original draft (lead), writing – review and editing (lead). **Sydney A. Almgren:** conceptualization (equal), data curation (supporting), formal analysis (supporting), writing – original draft (supporting), writing – review and editing (supporting). **J. Andrés López:** conceptualization (equal), formal analysis (supporting), funding acquisition (equal), project administration (equal), writing – review and editing (supporting). **Jessica R. Glass:** conceptualization (equal), funding acquisition (equal), project administration (equal), visualization (supporting), writing – review and editing (supporting).

## Conflicts of Interest

The authors declare no conflicts of interest.

## Data Availability

Raw sequence reads were deposited in the NCBI SRA under BioProject accession PRJNA1356517. A detailed description of data assembly, filtering, and analysis is available at https://github.com/letimm/pacific‐herring_lcWGS.

## Appendix A

**TABLE A1:** Metadata associated with sample collections, including the collection date, coordinates, region, and spawning status of the site. Individual size data, including length (in cm) and weight (in grams) is also reported.

Barcode	Date	Latitude_DD	Longitude_DD	MarineRegion	Spawning	Length_cm	weight_g
160128	4/10/2023	57.32	−152.99	Kodiak–Kiliuda	Spawning	23	168
160133	4/10/2023	57.32	−152.99	Kodiak–Kiliuda	Spawning	21.5	169
160134	4/10/2023	57.32	−152.99	Kodiak–Kiliuda	Spawning	23.5	215
160138	4/10/2023	57.32	−152.99	Kodiak–Kiliuda	Spawning	24	220
160139	4/10/2023	57.32	−152.99	Kodiak–Kiliuda	Spawning	24	253
160151	4/10/2023	57.32	−152.99	Kodiak–Kiliuda	Spawning	23.5	215
160160	4/10/2023	57.32	−152.99	Kodiak–Kiliuda	Spawning	25.5	264
160161	4/10/2023	57.32	−152.99	Kodiak–Kiliuda	Spawning	24.5	230
160162	4/10/2023	57.32	−152.99	Kodiak–Kiliuda	Spawning	23	203
160164	4/10/2023	57.32	−152.99	Kodiak–Kiliuda	Spawning	25	269
160203	4/22/2023	57.81	−153.51	Kodiak–Uganik	Spawning	19.5	90
160206	4/22/2023	57.81	−153.51	Kodiak–Uganik	Spawning	22	156
160210	4/22/2023	57.81	−153.51	Kodiak–Uganik	Spawning	19	114
160211	4/22/2023	57.81	−153.51	Kodiak–Uganik	Spawning	NA	NA
160214	4/22/2023	57.81	−153.51	Kodiak–Uganik	Spawning	18	63
160221	4/22/2023	57.81	−153.51	Kodiak–Uganik	Spawning	20	103
160222	4/22/2023	57.81	−153.51	Kodiak–Uganik	Spawning	19.5	92
160223	4/22/2023	57.81	−153.51	Kodiak–Uganik	Spawning	18.5	104
160224	4/22/2023	57.81	−153.51	Kodiak–Uganik	Spawning	19	86
160225	4/22/2023	57.81	−153.51	Kodiak–Uganik	Spawning	17.5	74
160275	7/30/2023	55.29	−160.45	Popof Island	Non‐spawning	23.5	198
160280	7/30/2023	55.29	−160.45	Popof Island	Non‐spawning	23	223
160281	7/30/2023	55.29	−160.45	Popof Island	Non‐spawning	23	188
160284	7/30/2023	55.29	−160.45	Popof Island	Non‐spawning	23.5	215
160285	7/30/2023	55.29	−160.45	Popof Island	Non‐spawning	25.5	275
160286	7/30/2023	55.29	−160.45	Popof Island	Non‐spawning	22	180
160287	7/30/2023	55.29	−160.45	Popof Island	Non‐spawning	23	231
160288	7/30/2023	55.29	−160.45	Popof Island	Non‐spawning	23	266
160289	7/30/2023	55.29	−160.45	Popof Island	Non‐spawning	22	167
160290	7/30/2023	55.29	−160.45	Popof Island	Non‐spawning	21	157
160291	7/30/2023	55.29	−160.45	Popof Island	Non‐spawning	22.5	177
160297	7/30/2023	55.29	−160.45	Popof Island	Non‐spawning	24	233
160299	7/30/2023	55.29	−160.45	Popof Island	Non‐spawning	24	201
160300	7/30/2023	55.29	−160.45	Popof Island	Non‐spawning	23	190
160301	7/30/2023	55.29	−160.45	Popof Island	Non‐spawning	23	172
160306	7/30/2023	55.29	−160.45	Popof Island	Non‐spawning	22.5	118
160312	7/30/2023	55.29	−160.45	Popof Island	Non‐spawning	22.5	201
160313	7/30/2023	55.29	−160.45	Popof Island	Non‐spawning	21.5	175
160315	7/30/2023	55.29	−160.45	Popof Island	Non‐spawning	24	227
160316	7/30/2023	55.29	−160.45	Popof Island	Non‐spawning	26.5	284
CB51	7/16/1999	53.95	−166.42	Constantine Bay	Non‐spawning	NA	NA
CB52	7/16/1999	53.95	−166.42	Constantine Bay	Non‐spawning	NA	NA
CB61	7/16/1999	53.95	−166.42	Constantine Bay	Non‐spawning	NA	NA
CB62	7/16/1999	53.95	−166.42	Constantine Bay	Non‐spawning	NA	NA
CB63	7/16/1999	53.95	−166.42	Constantine Bay	Non‐spawning	NA	NA
CB68	7/16/1999	53.95	−166.42	Constantine Bay	Non‐spawning	NA	NA
CB69	7/16/1999	53.95	−166.42	Constantine Bay	Non‐spawning	NA	NA
CB70	7/16/1999	53.95	−166.42	Constantine Bay	Non‐spawning	NA	NA
CB71	7/16/1999	53.95	−166.42	Constantine Bay	Non‐spawning	NA	NA
CB74	7/16/1999	53.95	−166.42	Constantine Bay	Non‐spawning	NA	NA
CB75	7/16/1999	53.95	−166.42	Constantine Bay	Non‐spawning	NA	NA
CB78	7/16/1999	53.95	−166.42	Constantine Bay	Non‐spawning	NA	NA
CB83	7/16/1999	53.95	−166.42	Constantine Bay	Non‐spawning	NA	NA
CB87	7/16/1999	53.95	−166.42	Constantine Bay	Non‐spawning	NA	NA
CB88	7/16/1999	53.95	−166.42	Constantine Bay	Non‐spawning	NA	NA
CB89	7/16/1999	53.95	−166.42	Constantine Bay	Non‐spawning	NA	NA
CB90	7/16/1999	53.95	−166.42	Constantine Bay	Non‐spawning	NA	NA
CB92	7/16/1999	53.95	−166.42	Constantine Bay	Non‐spawning	NA	NA
CB93	7/16/1999	53.95	−166.42	Constantine Bay	Non‐spawning	NA	NA
CB94	7/16/1999	53.95	−166.42	Constantine Bay	Non‐spawning	NA	NA
PM100	5/15/1996	55.99	−160.58	Port Moller	Spawning	NA	NA
PM51	5/15/1996	55.99	−160.58	Port Moller	Spawning	NA	NA
PM53	5/15/1996	55.99	−160.58	Port Moller	Spawning	NA	NA
PM54	5/15/1996	55.99	−160.58	Port Moller	Spawning	NA	NA
PM58	5/15/1996	55.99	−160.58	Port Moller	Spawning	NA	NA
PM60	5/15/1996	55.99	−160.58	Port Moller	Spawning	NA	NA
PM62	5/15/1996	55.99	−160.58	Port Moller	Spawning	NA	NA
PM63	5/15/1996	55.99	−160.58	Port Moller	Spawning	NA	NA
PM67	5/15/1996	55.99	−160.58	Port Moller	Spawning	NA	NA
PM73	5/15/1996	55.99	−160.58	Port Moller	Spawning	NA	NA
PM74	5/15/1996	55.99	−160.58	Port Moller	Spawning	NA	NA
PM76	5/15/1996	55.99	−160.58	Port Moller	Spawning	NA	NA
PM82	5/15/1996	55.99	−160.58	Port Moller	Spawning	NA	NA
PM84	5/15/1996	55.99	−160.58	Port Moller	Spawning	NA	NA
PM86	5/15/1996	55.99	−160.58	Port Moller	Spawning	NA	NA
PM87	5/15/1996	55.99	−160.58	Port Moller	Spawning	NA	NA
PM92	5/15/1996	55.99	−160.58	Port Moller	Spawning	NA	NA
PM93	5/15/1996	55.99	−160.58	Port Moller	Spawning	NA	NA
PM95	5/15/1996	55.99	−160.58	Port Moller	Spawning	NA	NA
PM97	5/15/1996	55.99	−160.58	Port Moller	Spawning	NA	NA
417767	5/3/2022	58.844	−159.65	Togiak	Spawning	23.4	179.5
417824	5/3/2022	58.844	−159.65	Togiak	Spawning	24	173
417825	5/3/2022	58.844	−159.65	Togiak	Spawning	26.2	237
417828	5/3/2022	58.844	−159.65	Togiak	Spawning	24.5	223
417829	5/3/2022	58.844	−159.65	Togiak	Spawning	24.6	214
417830	5/3/2022	58.844	−159.65	Togiak	Spawning	26.2	267
417831	5/3/2022	58.844	−159.65	Togiak	Spawning	25.5	251
417832	5/3/2022	58.844	−159.65	Togiak	Spawning	26.3	292
417835	5/3/2022	58.844	−159.65	Togiak	Spawning	26.3	271
417840	5/3/2022	58.844	−159.65	Togiak	Spawning	25.7	272
417842	5/3/2022	58.844	−159.65	Togiak	Spawning	27.5	353
417846	5/3/2022	58.844	−159.65	Togiak	Spawning	25.2	235
417847	5/3/2022	58.844	−159.65	Togiak	Spawning	26.4	308
417848	5/3/2022	58.844	−159.65	Togiak	Spawning	26.2	302
417849	5/3/2022	58.844	−159.65	Togiak	Spawning	26.7	297
417851	5/3/2022	58.844	−159.65	Togiak	Spawning	28.5	337
417854	5/3/2022	58.844	−159.65	Togiak	Spawning	26	261
417861	5/3/2022	58.844	−159.65	Togiak	Spawning	27.5	300
418727	5/3/2022	58.844	−159.65	Togiak	Spawning	26.5	290
418730	5/3/2022	58.844	−159.65	Togiak	Spawning	25	264
418852	3/30/2023	60.5257	−146.0005	Cordova	Spawning	17.6	74
418854	3/30/2023	60.5257	−146.0005	Cordova	Spawning	16.6	62
418858	3/30/2023	60.5257	−146.0005	Cordova	Spawning	18	98
418859	3/30/2023	60.5257	−146.0005	Cordova	Spawning	19.9	129
418861	3/30/2023	60.5257	−146.0005	Cordova	Spawning	19.4	116
418863	3/30/2023	60.5257	−146.0005	Cordova	Spawning	17.6	81
418869	3/30/2023	60.5257	−146.0005	Cordova	Spawning	18.9	46
418870	3/30/2023	60.5257	−146.0005	Cordova	Spawning	16	44
418878	3/30/2023	60.5257	−146.0005	Cordova	Spawning	18.6	109
418944	3/30/2023	60.5257	−146.0005	Cordova	Spawning	18	88
418962	3/30/2023	60.5257	−146.0005	Cordova	Spawning	16.4	62
418977	3/30/2023	60.5257	−146.0005	Cordova	Spawning	18.1	86
418990	3/30/2023	60.5257	−146.0005	Cordova	Spawning	17	63
418997	3/30/2023	60.5257	−146.0005	Cordova	Spawning	16.7	68
418998	3/30/2023	60.5257	−146.0005	Cordova	Spawning	19.7	111
419002	3/30/2023	60.5257	−146.0005	Cordova	Spawning	19	100
419004	3/30/2023	60.5257	−146.0005	Cordova	Spawning	16.8	67
419007	3/30/2023	60.5257	−146.0005	Cordova	Spawning	17.9	92
419011	3/30/2023	60.5257	−146.0005	Cordova	Spawning	21	145
419014	3/30/2023	60.5257	−146.0005	Cordova	Spawning	17	75

**TABLE A2:** Computational resources and custom scripts.

Tool	Version	Type	Sources
ade4	1.7–19	R package	Chessel et al. (2004); Dray and Dufour (2007); Dray et al. 2007; Bougeard and Dray (2018); Thioulouse et al. (2018)
adegenet	2.1.5	R package	Jombart (2008); Jombart and Ahmed (2011)
dismo	1.3–5	R package	Hijmans et al. (2021)
ape	5.6–2	R package	Paradis and Schliep (2019)
dplyr	1.1.2	R package	Wickham et al. (2023)
ggOceanMaps	1.3.4	R package	Vihtakari (2023)
ggplot2	3.4.4	R package	Wickham (2016)
ggpmisc	0.5.4–1	R package	Aphalo (2023)
ggpubr	0.4.0	R package	Kassambara (2020)
ggspatial	1.1.10	R package	Dunnington (2025)
gridExtra	2.3	R package	Auguie et al. (2017)
gtools	3.9.2	R package	Warnes et al. (2021)
hierfstat	0.5–10	R package	Goudet (2005)
label.switching	1.8	R package	Papastamoulis (2015)
pegas	1.1	R package	Paradis (2010)
pophelper	2.3.1	R package	Francis (2017)
poppr	2.9.3	R package	Kamvar et al. (2014); Kamvar et al. (2015)
RColorBrewer	1.1.3	R package	Neuwirth (2022)
readr	2.1.5	R package	Wickham, Hester, and Bryan (2024)
readxl	1.4.3	R package	Wickham and Bryan (2023)
remotes	2.4.2	R package	Csárdi et al. (2021)
reshape2	1.4.4	R package	Wickham (2007)
rgdal	1.5–31	R package	Bivand (2022)
runner	0.4.3	R package	Kałędkowski (2023)
scales	1.2.1	R package	Wickham and Seidel (2023)
stringr	1.5.0	R package	Wickham (2022)
tidyr	1.3.1	R package	Wickham, Vaughan, and Girlich (2024)
tidyverse	2.0.0	R package	Wickham et al. (2019)
convert beagle to FASTA		Script	https://github.com/letimm/WGSfqs‐to‐genolikelihoods/blob/main/scripts/beagle2fasta.py
fst significance test		Script	https://github.com/letimm/WGSfqs‐to‐genolikelihoods/blob/main/fst_significance_test.py
genotype heatmap		Script	See “ebs_chr7region_genoheatmap” in https://github.com/letimm/pacific‐herring_lcWGS/blob/main/RMarkdown_htmls/pacific_herring_summary_202509.Rmd
mean individual sequencing coverage		Script	https://github.com/letimm/WGSfqs‐to‐genolikelihoods/blob/main/mean_cov_ind.py
bamutil	1.0.15	Software	https://genome.sph.umich.edu/wiki/BamUtil
GIMP	2.10.38	Software	https://www.gimp.org/
ngsLD	1.2.1	Software	https://github.com/fgvieira/ngsLD
ngsParalog	1.3.4	Software	https://github.com/tplinderoth/ngsParalog
Picard	2.26.6	Software	http://broadinstitute.github.io/picard/
R	4.2.0	Software	R Core Team (2021)

**TABLE A3:** Pairwise weighted *F*_*ST*_ values between regions under the three‐population scenario, where Popof Island (PI), northern Gulf of Alaska (nGOA), and the eastern Bering Sea and Aleutian Islands (eBSAI) are analyzed as separate regions.

	eBSAI	nGOA	PI
eBSAI	—	0.193	0.151
nGOA	< 0.001	—	0.022
PI	< 0.001	0.105	—

*Note: p*‐values are provided below the diagonal.
