# Correction to “Biogeographic Structure and Mitonuclear Discordance Reveal Cryptic Diversity in Pacific Herring (*Clupea pallasii*)”

**DOI:** 10.1002/ece3.74324

**Published:** 2026-09-04

Timm, L. E. , S. A. Almgren , J. A. López , and J. R. Glass . 2025. “Biogeographic Structure and Mitonuclear Discordance Reveal Cryptic Diversity in Pacific Herring (*Clupea pallasii*).” Ecology and Evolution 15, no. 11: e72452. 10.1002/ece3.72452.41282327 PMC12638094

The first paragraph of section 4.2, “Adaptation and Cryptic Diversity With Gene Flow in the Eastern Bering Sea,” states that “Only one genomic region was identified as differentiating between lineages and as being under selection: this gene region corresponded to *tmem8b*, syn. nasopharyngeal carcinoma‐associated gene 6 (*ngx6*).” Due to an error in individual cluster assignment, genomic variation associated with clustering in the eastern Bering Sea was localized to a region of the *tmem8b* gene. Although the differentiation we describe at this locus is valid and correct, it does not explain the clustering in the eastern Bering Sea PCA. Upon reassigning individuals to clusters, we identified two large structural variants (SVs) that dominate the second half of chromosome 7 and the second half of chromosome 12 (Figure 6).

**FIGURE 6:**
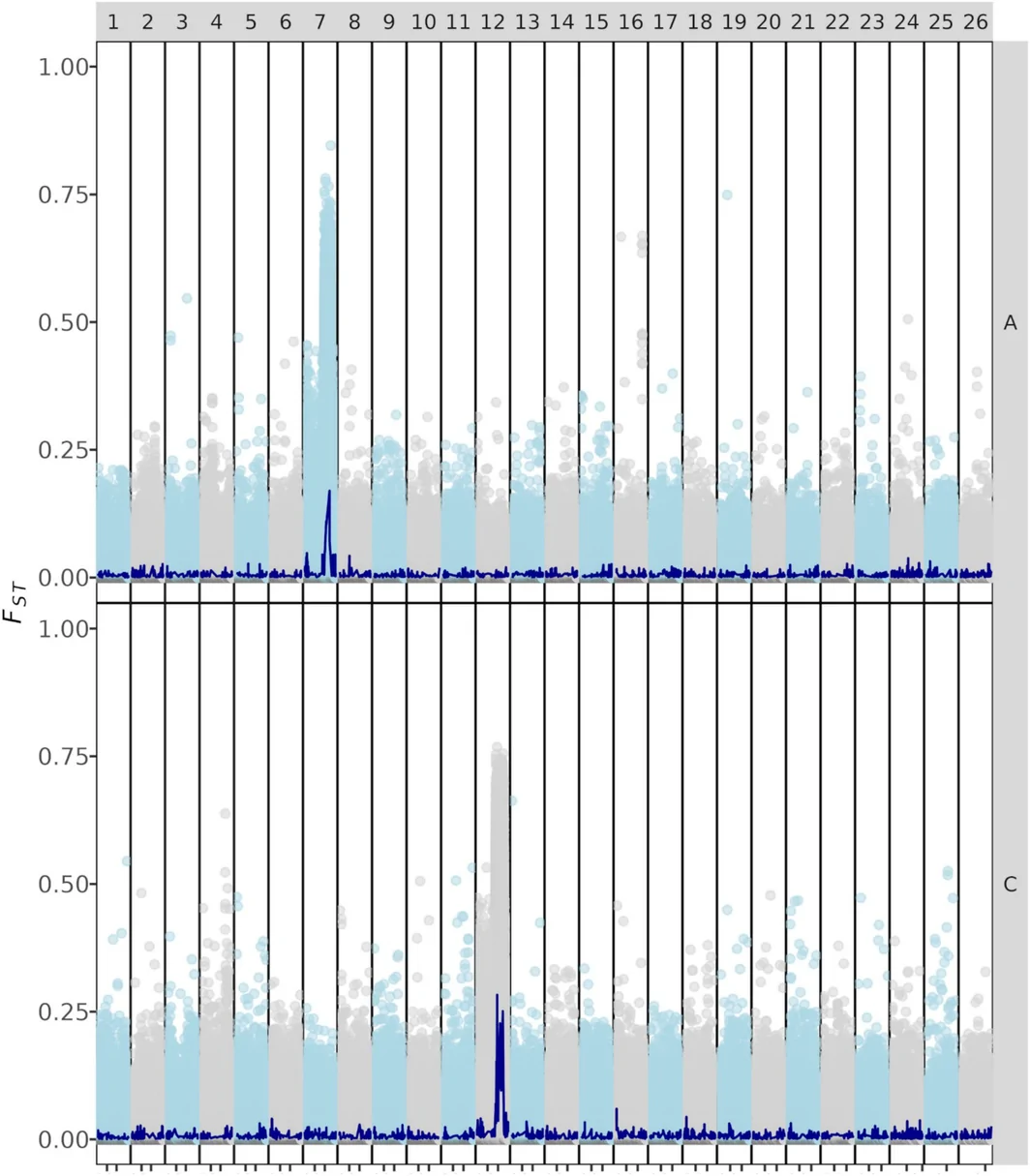
Manhattan plot of *FST* values calculated from SNPs across the genome comparing cluster B to cluster A (top) and cluster C (bottom), identified in the PCA (Figure 1C). Genomic positions on each chromosome are marked every 10 Mb.

We apologize for this error.

